# Age-specific information resources to address the needs of young people with stroke: a scoping review protocol

**DOI:** 10.1186/s13643-022-02147-4

**Published:** 2022-12-19

**Authors:** U. Gopaul, M. Charalambous, S. Thilarajah, L. K. Kwah, S. Chapman, M. Bayley, M. Demers

**Affiliations:** 1 The KITE Research Institute, Toronto Rehabilitation Institute, Toronto, Canada; 2 Faculty of Science and Medicine, University of Fribourg, Fribourg, Switzerland; 3 Rehabilitation Sciences Department, Cyprus University of Technology, Limassol, Cyprus; 4 Department of Physiotherapy, Singapore General Hospital, Bukit Merah, Singapore; 5 Health and Social Sciences Cluster, Singapore Institute of Technology, Dover, Singapore; 6 University of Virginia Comprehensive Stroke Center, Charlottesville, USA; 7 Division of Biokinesiology and Physical Therapy, University of Southern California, Los Angeles, USA

## Abstract

**Background and aims:**

Young people with stroke (YPwS) persistently experience challenges with disability, social reintegration, employment, and financial stability to provide for themselves and their families. The aims of this scoping review are to (1) identify and collate information resources for YPwS and evidence-based self-managements programs and (2) identify gaps in age-specific resources available for YPwS after traditional rehabilitation services have ended and/or who are returning to live in the community.

**Methods:**

We will include both qualitative and quantitative studies, including all study designs. Participants will be community-dwelling adults aged between 18 and 65 years with a clinical diagnosis of stroke. We will include information resources and evidence-based self-managements programs for YPwS. Search terms will include stroke, young people, and community dwelling. We will search electronic databases such as MEDLINE. The reference lists of included studies, systematic reviews, and stroke guidelines and stroke-specific websites will also be searched. We will also contact Stroke Support Organizations and international/national allied health professional organisations to gather information resources about YPwS. We will also conduct a comprehensive environmental scan of additional resources using the search engine Google.

The titles, abstracts, full-text articles, and contents of the resources identified by the search will be assessed against the inclusion and exclusion criteria to identify potentially relevant resources.

**Results and conclusions:**

Existing resources and self-management programs will be collated and categorized according to the type of needs addressed such as physical, emotions, activities of daily living, information, relationships, and social needs as well as the key gaps identified.

**Supplementary Information:**

The online version contains supplementary material available at 10.1186/s13643-022-02147-4.

## Background

Young people with stroke (YPwS) face many physical, emotional, and psychosocial challenges, with reports of 44% being depressed, 43% not returning to work, and 28% rating quality of life as poor or worse than death [1]. Despite intensive rehabilitation, YPwS are often discharged from institutional care with persistent impairments such as motor movement and sensory losses, balance instability, gait deficits, communication difficulties, cognitive impairments, and challenges with everyday functionality [2–7]. The complex interaction between these impairments contributes to significant dependency in activities of daily living (ADL) and limited participation in community activities in the long term [8].

YPwS persistently experience challenges with social reintegration, employment, and financial stability to provide for themselves and their families [9, 10]. YPwS experience increased burden of disability-adjusted life years due to early disability, reduced productivity, increased disability claim, and significant out-of-pocket healthcare costs [1]. They also face marital issues, including sexual dysfunction [11], and limited opportunities for social participation [12]. Drawing from previous work on the needs of YPwS, diverse unmet physical, emotional, communication, cognitive, psychosocial, and professional needs were identified [13–15]. A recent international survey reported that face-to-face contact with a healthcare professional, succinct list of tips, and peer support can help to address unmet needs [16]. Various methods of meeting YPwS needs outside the traditional healthcare setting or face-to-face interaction with clinicians were identified, with varying preferences between subgroups based on demographic attributes [16]. While professional guidance with a healthcare professional is valued, it often remains limited or unavailable after discharge [17]. Currently, information resources and self-management programs to support the unique needs of this population are not centralized and can be challenging to find for community rehabilitation professionals, YPwS, and their family. For example, several online resources identified in the literature to support YPwS [18] are no longer active. This limits the access to up-to-date information on self-management strategies to manage poststroke psychosocial challenges.

### Aim and objectives

The overarching aim of this scoping review is to examine the information resources to address the needs of YPwS, their families, and caregivers. The objectives are to (1) identify, collate, and appraise the quality of information resources for YPwS and evidence-based self-management programs, (2) identify gaps in age-specific resources available for YPwS after traditional rehabilitation services have ended and/or who are returning to live in the community, and (3) conduct a consultation exercise to provide the opinion of YPwS about the resources identified and gaps identified in meeting their needs.

### Research question

This review is guided by the following research questions:

1. What information resources (e.g., information pamphlets, fact sheets, list of tips, videos, podcasts, smartphone applications, websites, evidence-based self-management programs) about self-management have been proposed to address the needs of YPwS, family, and caregivers? What is the quality of the information resources identified?
2. What are the gaps in resources available for YPwS, family, and caregivers after traditional rehabilitation has ended?
3. What are the views of YPwS about existing resources and gaps identified in meeting their needs?

## Methods

We will conduct a systematic scoping review of the scientific and gray literature to identify resources and self-management programs to meet the needs of YPwS, their family, and caregivers. The scoping review protocol was developed based on the Arksey and O’Malley framework [19] and the recommendations by Levac et al. [20]. It follows the PRISMA Extension for Scoping Reviews (Prisma-ScR: Preferred Reporting Items for Systematic Reviews and Meta-Analyses extension for Scoping Review) guidelines to increase methodological transparency [21]. This scoping review protocol was registered on Open Science Framework [22].

### Inclusion and exclusion criteria

#### Study design

We will include both qualitative and quantitative studies, including all study designs, except reviews and meta-analyses, as well as paper- and web-based resources.

#### Study population

Participants will be community-dwelling adults aged between 18 and 65 years with a clinical diagnosis of stroke and their caregivers. We will include studies that also recruited participants with other neurological disorders if the data on stroke subjects can be extracted from the data of non-stroke subjects (i.e., data from different groups should not be pooled). The types of resources or studies will include information pamphlets/sheets, fact sheets, list of tips, handouts, website, flyers, workbook, handbook, videos, podcasts, smartphone applications, home-based programs, and evidence-based self-management programs. Resources written in English, French, Greek, Dutch, Arabic, Portuguese, Spanish, and Chinese or any other language for which translation can be obtained will be included.

Resources or studies that include children aged less than 18 years, older adults aged more than 65 years, and individuals with neurological disorders other than stroke will be excluded. Personal resources such as testimonies and personal blogs will be excluded as these have been addressed in a recent study [23].

### Identify relevant studies

#### Scientific literature

The databases MEDLINE (Ovid), Excerpta Medica Database (Embase), Cumulative Index to Nursing and Allied Health Literature (CINAHL), Allied and Complementary Medicine Database (AMED), Joanna Briggs Institute Evidence-Based Practice Database, and Cochrane Central Register of Controlled Trials (CENTRAL) will be searched up to the year 2022. The electronic search strategy will be developed based on the population-concept-context scoping review framework recommended by the Joanna Briggs Institute [24, 25] with the assistance of a medical librarian using Medical Subject Heading (MeSH) and individual keywords illustrated in Table 1.

**Table 1:** Population-concept-context framework for the electronic search strategy

Framework	Theme	Key terms	Keywords
Population	Young adults with stroke, their families, or caregivers	Stroke	Stroke or CVA
		Young adults (18–65 years)	Young adult or patient or survivor
		Family, caregivers	Family or caregiver or carer
Concept	Information	Information, education resources, self-management	Needs or education or support or information or resources or publication
			Self-care, self-management, self-rehabilitation
Context	Community based	Long-term stroke care	Long-term stroke care or poststroke care or post discharge or post rehabilitation
		Community	Community dwelling or community based or home based

The reference lists of included studies, reviews, and guidelines will be searched by hand to identify other relevant publications. To optimize the search for published literature, we will also search the Internet for additional reviews as well as national guidelines worldwide.

#### Gray literature

We will directly email stroke support organizations worldwide via the World Stroke Organization (e.g., Singapore National Stroke Association), stroke-related nonprofit organizations (e.g., World Heart and Stroke Foundation), and national and international allied health professional organizations (e.g., World Physiotherapy and World Occupational Therapy) and review their websites to obtain their information repository for young people with stroke.

We will also conduct a comprehensive web search in a predetermined time and date using the most popular search engine, Google (Google.com, Mountain View, CA, USA) which represents 87–92% of all search engines worldwide [26, 27]. A web search will be used to identify relevant web-based information for young people with stroke and to identify references to non-web-based information or unpublished work. We will execute the search using the following keyword strings: (1) health/(wellness OR well-being) literacy, (2) health/(wellness OR well-being) education, and (3) community literacy. To ensure a comprehensive search, efforts will be made to obtain any relevant documents. This may involve searching other sections of a multipage website and/or contacting the developers of the resources directly to request further information. Adhering to a methodology utilized by the Canadian Institute for Health Information (CIHI), we will include only the first 10 search engine result pages, consisting of the first 100 results [28].

We will also conduct a web search for websites with information intended for young people with stroke. Websites will be excluded if the literature is intended for health professionals or researchers and scientific journals or are used for marketing purposes. The URLs of eligible websites will be manually extracted, using copy and paste function, into an excel database.

Five reviewers (UG, MD, MC, ST, LKK) will conduct the search, and results from literature searches will be imported into a citation manager, and duplicates will be removed (Bramer et al., 2016). We will document the searches, including the full search strategy, the databases searched, and the search date. Data from all sources will be abstracted and stored in EndNote X9 software.

### Study selection

Two review authors (UG and MD) will independently screen the titles and abstracts of the articles displayed by the search against the inclusion and exclusion criteria to identify potentially relevant studies. Irrelevant studies will be discarded. Abstracts of the remaining studies will be assessed against the inclusion criteria by two review authors (UG and MD) and independently categorized as “possibly relevant” and “definitely irrelevant.” If abstracts were ranked as “definitely irrelevant” by both review authors, these studies will be excluded at this stage. Full-text articles classified as “possibly relevant” will be retrieved for all selected citations and screened for eligibility using standardized criteria in Covidence. These studies will be independently ranked as “include,” “exclude,” or “unsure.” Studies classified as “unsure” by both review authors will be reviewed by a third reviewer. If there is a disagreement between review authors, or a decision could not be reached, consensus will be made through discussion. A third reviewer will be included to resolve disagreement, and Kappa coefficients will be reported, as necessary.

### Charting the data

Four reviewers will perform data extraction with verified by two additional reviewers. A data-charting form will be developed and used to extract data from each publication. Elements of data extraction will include study design, data source, organization (academic center, stroke support organizations, nonprofit organizations, allied health organizations, industry), country of origin, resource type (information pamphlets/sheets, fact sheets, list of tips, handouts, website, flyers, workbook, handbook, videos, podcasts, smartphone applications, information, protocols, strategies, interventions, evidence-based self-management programs), type of needs addressed (physical, emotional, communication, cognitive, psychosocial and professional needs), information/program contents (definition of stroke, stroke pathophysiology, mechanisms and risk factors, secondary prevention, pain and fatigue management, scope and strategies of rehabilitation, community services, falls prevention, finance management, diet, mindfulness, drugs management), target population (YPwS, family, caregivers), citation, or link to source material. For self-management programs, length, format, setting, and evidence of effectiveness will also be extracted. Missing information from the publications will be registered, and authors will be contacted for additional information.

#### Assessment of the methodological quality of resources

For studies identified, the Downs and Black checklist [29] will be used to assess the methodological quality of randomized and non-randomized studies. The Downs and Black checklist evaluates 27 items relating to the reporting of findings, external validity, internal validity (bias and confounding), and the statistical power. The scores range from 0 to 28, and the corresponding quality levels are graded as follows: excellent (26–28), good (20–25), fair (15–19), and poor (≤ 14) [30]. The Oxford Centre for Evidence-Based Medicine guidelines [31] will be used to evaluate the “Levels of Evidence” (1a–5) of the types of study designs included. Each written patient information from resources such as pamphlets or websites identified will be reviewed by two authors using the DISCERN questionnaire. DISCERN is a valid and reliable instrument for analyzing written consumer health information [32]. It consists of 16 questions categorized in three sections: (a) section 1 (questions 1 to 8) evaluates reliability, dependability, and trustworthiness of a source of information, (b) section 2 (questions 9 to 15) evaluates the quality of information about treatment choices, (c) and section 3 (question 16) evaluates overall quality. The rating scale ranges from 1 to 5, where 1 = definite no and 5 = definite yes [33]. The tool has been used widely to evaluate the quality of patient information in healthcare (number of citations by 1389). Good quality patient information based on best and most up-to-date scientific evidence will be prioritized.

### Collating, summarizing, and reporting results

Existing resources and self-management programs will be collated and categorized according to the type of needs addressed. The types of needs will be classified as follows: physical functions (movement, swallowing, pain, general health, ambulation), cognitive functions (concentration, memory, executive functions), emotions (mood, depression, anxiety, sense of feeling respected, identity), information and education (poststroke care and rehabilitation, stroke type, cause, prevention, recovery, secondary prevention and self-management), activities of daily living (household chores, returning to work and school, driving), relationships (impact on close relationships, parenting, intimacy and sexuality, family planning), social participation (isolation, support from family and friends, community reintegration), and rehabilitation and care (physiotherapy, occupational therapy, speech and language therapy, nursing care, home care).

Narrative description of the data will summarize and discuss the resources available to assist YPwS in their relevant communities, the key gaps in resources tailored to YPwS, and conclusions organized around the aims of this review. Where possible, tables and figures will also be used to present the results.

### Consultation exercise

Researchers will meet with a sample of ten YPwS (aged between 18 and 55 years old) in a one-on-one meeting. During the meeting, the researcher will present the results of the scoping review in lay terms. Then, we will use semi-structured interviews to ask YPwS about their opinion about the current resources offered, whether they meet their needs and additional gaps perceived. The semi-structured interviews will be videotaped and transcribed verbatim. Transcripts will be analyzed using inductive thematic content analysis according to Braun and Clarke (2006) [34].

## Expected results

**Fig. 1:**
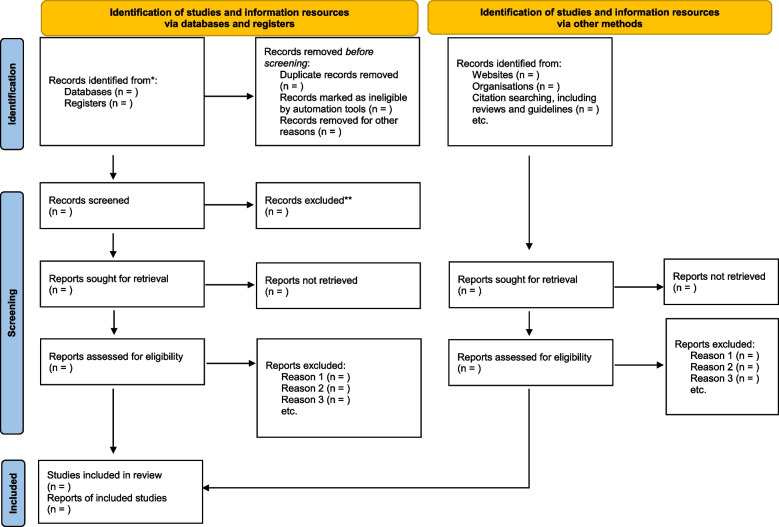
PRISMA 2020 flow diagram for scoping review process

We have piloted our electronic search strategy in MEDLINE (Additional file 1: Appendix A) which yielded 1475 titles and abstract after removal of duplicates. The preliminary web search for additional published literature has identified 25 consensus study and reviews (Additional file 2: Appendix B) and 54 guidelines worldwide (Additional file 3: Appendix C) to be assessed against the inclusion criteria. Our search has also identified 17 stroke-support and stroke-related nonprofit organisations (Additional file 4: Appendix D) and 289 allied professionals’ associations to be contacted for information sources for YPwS (Additional file 5: Appendix E) which may contain information for YPwS. The identification of studies and resources from databases and from other sources will be reported in the PRISMA 2020 flow diagram template [35] (Fig. 1).

## Strengths and limitations

This scoping review focuses on age-specific resources for YPwS who have been discharged from rehabilitation and who live in the community. Additionally, we will use an extensive search strategy to identify published as well as unpublished information resources worldwide. This review will include translations of resources published in non-English languages, which are otherwise neglected. This will help identify additional key data which will reduce bias and improve the quality of this review. The reviewing of national guidelines across the world as well as the environmental scan of resources from national and international stroke and rehabilitation organisations will ensure a comprehensive search for information resources from countries with varied sources of income. Although not mandatory, the consultation exercise with YPwS as partners in their recovery and community reintegration to normal living is crucial in informing and validating findings from the review. This exercise may also reveal further unmet needs unaddressed so far in the literature. One limitation of this study is that even though we will contact stroke-related organisations, we might lack access to printed stroke education resources for YPwS that are often widely used by independent or local clinics, especially in low-resource settings with poor Internet access or low health e-literacy.

## Discussion and dissemination

The proposed scoping review aims to identify the information resources and self-management programs targeted for YPwS. We anticipate that the findings from this study will highlight health-related needs addressed by traditional stroke care and rehabilitation as well as the non-health-related needs specific for YPwS living in the community that ought to be equally prioritized. These findings may contribute to the design of age-specific evidence-based educational and self-management programs to improve the overall well-being, quality of life, and community reintegration to normal living for YPwS. This scoping review can be used to inform future national and international clinical practice guidelines to address the specific needs of YPwS for community reintegration. The results may lead to centralizing existing resources that may not be accessible to YPwS, their families, and healthcare professionals.

The findings from the scoping review will be submitted for publication in a peer-reviewed journal. Moreover, the resources identified will be packaged into a user-friendly format to facilitate access to YPwS, their families, and healthcare professionals. A repository of these resources, translated in multiple languages, will be hosted on the World Stroke Organization website for dissemination to a large audience.

## Supplementary Information

**Additional file 1: Appendix A.** Search strategy for Ovid MEDLINE(R) ALL <1946 to April, 11 2022; n= 1487 >.

**Additional file 2: Appendix B.** Reviews on information resources for young people with stroke.

**Additional file 3: Appendix C.** Stroke guidelines.

**Additional file 4: Appendix D.** Stroke -support and stroke-related Non-Profit Organisations websites.

**Additional file 5: Appendix E.** Allied Health Professional Organizations.
