# Supplementary material for: Age-specific information resources to address the needs of young people with stroke: a scoping review protocol
Source: Syst Rev. 2022 Dec 19;11:275. doi: 10.1186/s13643-022-02147-4 (PMC9761956; doi:10.1186/s13643-022-02147-4)
Supplement: Supplementary file 1 — Additional file 1: Appendix A. Search strategy for Ovid MEDLINE(R) ALL <1946 to April, 11 2022; n= 1487 >. [file 13643_2022_2147_MOESM1_ESM.docx]

**Appendix A. Search strategy for Ovid MEDLINE(R) ALL <1946 to April,11 2022; n=** 1487 **>**

1 (Stroke* or poststroke*).tw,kf.

2 exp Stroke Rehabilitation/ or exp Stroke/

3 (cerebrovascular adj1 accident*).tw,kf.

4 CVA*.tw,kf.

5 exp Cerebrovascular Disorders/

6 1 or 2 or 3 or 4 or 5

7 ((young or younger or working age) adj3 (patient* or survivor* or adult* or person*)).tw,kf.

8 limit 7 to ("young adult and adult (19-24 and 19-44)" or "middle age (45 to 64 years)")

9 exp Caregivers/

10 exp Family/

11 (Patient* or inpatient* or carer* or care?giver* or family).tw,kf.

12 8 or 9 or 10 or 11

13 ((Patient* or inpatient* or carer* or care?giver* or family) adj3 (support or need* or resource* or education or information or knowledge or counsel* or publication* or Program* or intervention* or material* or resource* or Book* or leaflet* or pack* or video* or tape* or telephone or manual* or advice)).tw,kf.

14 exp Health Education/

15 exp Health Promotion/

16 exp Patient Education as Topic/

17 exp Health Knowledge, Attitudes, Practice/

18 exp Telephone/

19 exp Pamphlets/

20 exp Books/

21 Manual*.mp.

22 exp Audiovisual Aids/

23 exp Counseling/

24 exp Tape Recording/

25 exp Video Recording/

26 13 or 14 or 15 or 16 or 17 or 18 or 19 or 20 or 21 or 22 or 23 or 24 or 25

27 ((care or rehab) and (community or long term or post stroke or post discharge)).tw,kf.

28 (community adj3 (dwelling or living)).tw,kf.

29 Communit*.tw,kf.

30 (independent adj3 living).tw,kf.

31 27 or 28 or 29 or 30

32 6 and 12 and 26 and 31

33 limit 32 to humans
