# Supplementary material for: Age-specific information resources to address the needs of young people with stroke: a scoping review protocol
Source: Syst Rev. 2022 Dec 19;11:275. doi: 10.1186/s13643-022-02147-4 (PMC9761956; doi:10.1186/s13643-022-02147-4)
Supplement: Supplementary file 2 — Additional file 2: Appendix B. Reviews on information resources for young people with stroke. [file 13643_2022_2147_MOESM2_ESM.docx]

**Appendix B. Reviews on information resources for young people with stroke**

| **Consensus study (n=1)** |
| --- |
| Singhal AB, Biller J, Elkind MS, Fullerton HJ, Jauch EC, Kittner SJ, et al. Recognition and management of stroke in young adults and adolescents. Neurology. 2013;81(12):1089-97. |
| **Narrative reviews (n= 10)** |
| Gilmore N, Katz DI, Kiran S. Acquired Brain Injury in Adults: A Review of Pathophysiology, Recovery, and Rehabilitation. Perspect ASHA Spec Interest Groups. 2021;6(4):714-27. |
| Graham JR, Pereira S, Teasell R. Aphasia and return to work in younger stroke survivors. Aphasiology. 2011;25(8):952-60. |
| McCarty JL, Leung LY, Peterson RB, Sitton CW, Sarraj A, Riascos RF, et al. Ischemic Infarction in Young Adults: A Review for Radiologists. Radiographics. 2019;39(6):1629-48. |
| Boot E, Ekker MS, Putaala J, Kittner S, De Leeuw FE, Tuladhar AM. Ischaemic stroke in young adults: a global perspective. J Neurol Neurosurg Psychiatry. 2020;91(4):411-7. |
| Yahya T, Jilani MH, Khan SU, Mszar R, Hassan SZ, Blaha MJ, et al. Stroke in young adults: Current trends, opportunities for prevention and pathways forward. Am J Prev Cardiol. 2020;3:100085. |
| Putaala J. Ischemic Stroke in Young Adults. Continuum (Minneap Minn). 2020;26(2):386-414. |
| Berkman SA, Song SS. Ischemic Stroke in the Young. Clin Appl Thromb Hemost. 2021;27:10760296211002274. |
| Teasell RW, McRae MP, Finestone HM. Social issues in the rehabilitation of younger stroke patients. Arch Phys Med Rehabil. 2000;81(2):205-9. |
| Chang WH, Sohn MK, Lee J, Kim DY, Lee SG, Shin YI, et al. Return to work after stroke: The KOSCO Study. J Rehabil Med. 2016;48(3):273-9. |
| Zawawi NSM, Aziz NA, Fisher R, Ahmad K, Walker MF. The Unmet Needs of Stroke Survivors and Stroke Caregivers: A Systematic Narrative Review. J Stroke Cerebrovasc Dis. 2020;29(8):104875. |
| **Scoping reviews (n= 3)** |
| Holloway A, Chandler C, Aviles Reinso L, Clarissa C, Putri A, Choi H, et al. Young Adults Rehabilitation Needs and Experiences following Stroke (YARNS): A review of digital accounts to inform the development of age-appropriate support and rehabilitation. J Adv Nurs. 2021. |
| Hathidara MY, Saini V, Malik AM. Stroke in the Young: a Global Update. Curr Neurol Neurosci Rep. 2019;19(11):91. |
| Krishnan S, Pappadis MR, Weller SC, Stearnes M, Kumar A, Ottenbacher KJ, et al. Needs of Stroke Survivors as Perceived by Their Caregivers: A Scoping Review. Am J Phys Med Rehabil. 2017;96(7):487-505. |
| **Systematic reviews (n= 11)** |
| Harris GM, Prvu Bettger J. Parenting after stroke: a systematic review. Top Stroke Rehabil. 2018;25(5):384-92. |
| Turner-Stokes L, Pick A, Nair A, Disler PB, Wade DT. Multi-disciplinary rehabilitation for acquired brain injury in adults of working age. Cochrane Database Syst Rev. 2015;2015(12):Cd004170. |
| Eghdam A, Scholl J, Bartfai A, Koch S. Information and communication technology to support self-management of patients with mild acquired cognitive impairments: systematic review. J Med Internet Res. 2012;14(6):e159. |
| Daniel K, Wolfe CD, Busch MA, McKevitt C. What are the social consequences of stroke for working-aged adults? A systematic review. Stroke. 2009;40(6):e431-40. |
| Xu L, Pan X, Zhou C, Li J, Wang F. Long-term efficacy after closure of patent foramen ovale for ischemic neurological events in young adults: A systematic review and meta-analysis. Medicine (Baltimore). 2020;99(2):e18675. |
| Ekker MS, Jacob MA, van Dongen MME, Aarnio K, Annamalai AK, Arauz A, et al. Global Outcome Assessment Life-long after stroke in young adults initiative-the GOAL initiative: study protocol and rationale of a multicentre retrospective individual patient data meta-analysis. BMJ Open. 2019;9(11):e031144. |
| Cheng HY, Chair SY, Chau JP. The effectiveness of caregiver psychosocial interventions on the psychosocial wellbeing, physical health and quality of life of stroke family caregivers and their stroke survivors: A systematic review. JBI Libr Syst Rev. 2012;10(12):679-797. |
| Forster A, Brown L, Smith J, House A, Knapp P, Wright JJ, et al. Information provision for stroke patients and their caregivers. Cochrane Database Syst Rev. 2012;11(11):Cd001919. |
| Edwards JD, Kapoor A, Linkewich E, Swartz RH. Return to work after young stroke: A systematic review. Int J Stroke. 2018;13(3):243-56. |
| Lawrence M. Young adults' experience of stroke: a qualitative review of the literature. Br J Nurs. 2010;19(4):241-8. |
| Wray F, Clarke D. Longer-term needs of stroke survivors with communication difficulties living in the community: a systematic review and thematic synthesis of qualitative studies. BMJ Open. 2017;7(10):e017944. |
