# Supplementary material for: Age-specific information resources to address the needs of young people with stroke: a scoping review protocol
Source: Syst Rev. 2022 Dec 19;11:275. doi: 10.1186/s13643-022-02147-4 (PMC9761956; doi:10.1186/s13643-022-02147-4)
Supplement: Supplementary file 3 — Additional file 3: Appendix C. Stroke guidelines. [file 13643_2022_2147_MOESM3_ESM.docx]

**Appendix C: Stroke guidelines**

| **REGION: AFRICA (n= 5)** |
| --- |
| **CAMEROON** |
| The NWR best practices in stroke rehabilitation group. Best practice guidelines for the management and rehabilitation of stroke in the North West region of Cameroon 2013 [cited 2022 February, 3]. Available from: <http://icdr.utoronto.ca/wp-content/uploads/2015/05/Best-Practice-Guideline-Stroke-rehabilitation-for-NWR-Final-.pdf> |
| **NAMIBIA** |
| Ministry of Health and Social Services. Namibia standard treatment guidelines 2011 [cited 2022 February, 3]. Available from: <http://www.man.com.na/files/news/1501069447namibia-standard-treatment-guidelines.pdf>. |
| **SOUTH AFRICA**  Bryer A, Connor M, Haug P, Cheyip B, Staub H, Tipping B, et al. South African guideline for management of ischaemic stroke and transient ischaemic attack 2010: a guideline from the South African Stroke Society (SASS) and the SASS Writing Committee. S Afr Med J. 2010;100(11 Pt 2):747-78. |
| Bryer A, Connor MD, Haug P, Cheyip B, Staub H, Tipping B, et al. The South African Guideline for the Management of Ischemic Stroke and Transient Ischemic Attack: Recommendations for a Resource-Constrained Health Care Setting. International Journal of Stroke. 2011;6(4):349-54. |
| SA Stakeholder Reference Group. South African contextualised stroke rehabilitation guideline (SA-CSRG) 2019 [cited 2022 February, 3]. Available from: <http://icdr.utoronto.ca/wp-content/uploads/2015/05/Best-Practice-Guideline-Stroke-rehabilitation-for-NWR-Final-.pdf> |
| **REGION: ASIA (n= 15)** |
| **CHINA**  Zhao FY, Yue YY, Li L, Lang SY, Wang MW, Du XD, et al. Clinical practice guidelines for post-stroke depression in China. Braz J Psychiatry. 2018;40(3):325-34. |
| **INDIA**  Ministry of Health and Family Welfare India. ﻿Guidelines for Prevention and Management of Stroke (2019) [cited 2022, March 23]. Available from: <https://main.mohfw.gov.in/sites/default/files/Guidelines%20for%20Prevention%20and%20Managment%20of%20Stroke.pdf> |
| **KOREA** |
| Kim DY KY, Lee J, Chang WH, Kim MW, Pyun SB, et al. Clinical Practice Guideline for Stroke Rehabilitation in Korea. Brain Neurorehabilitation. 2017;10(1). |
| **MALAYSIA**  Ministry of Health Malaysia. Clinical Practice Guidelines Management of Ischemic Stroke 3^rd^ Edition 2020. Kuala Lumpur: Malaysian Society of Neurosciences; 2021 [cited 2022, March 23]. Available from: <https://www.moh.gov.my/moh/resources/Penerbitan/CPG/CARDIOVASCULAR/CPG_Management_of_Ischaemic_Stroke_3rd_Edition_2020_28.02_.2021_.pdf> |
| **MONGOLIA** |
| Ministry of Health of Mongolia. Management of patients with stroke: Rehabilitation, prevention and management of complications, and discharge planning: Ulaanbaatar; 2013 [cited 2022 February, 3]. Available from: <https://extranet.who.int/ncdccs/Data/MNG_D1_2.%20Rehabilitation%20guideline%20of%20Stroke.pdf>. |
| **OMAN** |
| Ministry of Health Sultanate of Oman. Acute Stroke Management Protocol (2020) [cited 2022, March 23]. Available from: <https://www.moh.gov.om/documents/17733/121232/Acute+stroke+management/405ccc8d-43c4-979a-c9c8-509e5ae36c89> |
| Ministry of Health Sultanate of Oman. Physiotherapy Procedure for Stroke Rehabilitation (2020) [cited 2022, March 23]. Available from: <https://www.moh.gov.om/documents/17733/4449443/Physiotherapy+Procedure+for+Stroke+Rehabilitation.pdf/f4159f88-9981-fa6c-9e82-db9dcb496814> |
| **PAKISTAN**  Kamal, A. K., Itrat, A., Naqvi, I., Khan, M., Channa, R., Khatri, I., Wasay, M. (2010). Ischemic stroke care - official guidelines from the Pakistan society of Neurology. Pakistan Journal of Neurological Sciences, 51(1), 38-43. Available from: <http://ecommons.aku.edu/pakistan_fhs_mc_med_med/128> |
| **PHILIPPINES** |
| Philippine Academy of Rehabilitation Medicine. Clinical Practice Guideline on Stroke Rehabilitation. Medicine PAoR, editor. Philippines: GoldenPages Publishing Company; 2017. |
| The Stroke Society of Philipinnes. SSP Handbook of Stroke: Guidelines, Prevention, Treatment and Rehabilitation. Sixth ed. Jr. AAR, editor. Philipinnes: GoldenPages Publishing Company; 2014. |
| **QATAR** |
| Ministry of Public Health Qatar. National Clinical Guideline: The Diagnosis and Management of Stroke and Transient Ischaemic Attack (2020) [cited 2022, March 23]. Available from: <https://www.moph.gov.qa/english/OurServices/eservices/Pages/Clinical-Guidelines.aspx> |
| **SINGAPORE** |
| Sithamparapillai GS. Stroke rehabilitation rehabilitation principles. The Singapore Family Physician. 2019;45(3). |
| \| **SOUTH KOREA** \| \| --- \| \| Rha J-H, Hong K-S, K-H. Y. Clinical practice guidelines for stroke. Seoul: Clinical Research Centre for Stroke; 2015. \| |
| \| **SRI LANKA** \| \| --- \| \| Ceylon College of Physicians. Management of stroke and transient ischemic attacks in adults: Clinical Guidelines 2017.Colombo:CCP;2017 [cited 2022, March 23]. Available from: <https://www.ccp.lk/wp-content/uploads/guidelines-ccp-stroke-guidelines-2017.pdf> \| |
| Gunaratne P RM, Peiris JB, Gubasekara H, Alibhoy AT, Senanayake B, et al. Management of stroke Colombo: Ministry of Health of Sri Lanka; 2015 [cited 2022 February, 3]. Available from: https://shri.lk/wp-content/uploads/2015/07/Management-of-Stroke.pdf |
| **REGION: AUSTRALASIA (n= 3)** |
| **AUSTRALIA/ NEW ZEALAND** |
| Clinical Centre for Research in Aphasia Rehabilitation. Aphasia rehabilitation best practice statements Brisbane2014 [cited 2022 February, 3]. Available from: <http://www.aphasiapathway.com.au/flux-content/aarp/pdf/2014-COMPREHENSIVE-FINAL-01-10-2014-1.pdf>. |
| National stroke foundation Australia. A Clinical Practice Guideline for Stroke Management 2010 [cited 2022 February, 3]. Available from: <https://www.pedro.org.au/wp-content/uploads/CPG_stroke.pdf>. |
| Stroke Foundation. Australian and New Zealand Clinical Guidelines for Stroke Management [updated 2021; cited 2022 February, 3]. Available from: <https://app.magicapp.org/#/guideline/5536> |
| **REGION: EUROPE (n= 16)** |
| **FRANCE** |
| Haute Autorité de Santé. Accident Vasculaire Cérébral: Méthodes de Rééducation de la Fonction Motrice Chez L'adulte 2012 [cited 2022 Februrary, 3]. Available from: <https://www.has-sante.fr/portail/upload/docs/application/pdf/201211/11irp01_reco_avc_methodes_de_reeducation.pdf>. |
| **GERMANY** |
| German Society for Neurorehabilitation. Motorische Therapien für die obere Extremität zur Behandlung des Schlaganfalls. S2e Leitlinie 2009 [cited 2022 February, 3]. Available from: <https://www.awmf.org/leitlinien/detail/ll/080-001.html>. |
| German Society for Neurorehabilitation. Rehabilitation der Mobilität nach Schlaganfall. S2e Leitlinie 2015 [cited 2022 February, 3]. Available from: <https://www.awmf.org/leitlinien/detail/ll/080-004.html>. |
| **IRELAND**  Irish Heart Foundation. Council for Stroke National Clinical Guidelines and Recommendations for the Care of People with Stroke and Transient Ischaemic Attack Revised Version (2010) [cited 2022, March 27]. Available from: <http://irishheart.ie/wp-content/uploads/2017/04/guidelines.pdf> |
| **NETHERLANDS** |
| Berns PEG JN, Boxum E, Nouwens F, van der Staaij MG, van Wessel S, et al. Speech therapy guideline. Diagnostics and treatment of aphasia in adults: Dutch Association for Speech Therapy and Phoniatrics; 2015 [cited 2022 February, 3]. |
| Steultjens EMJ, Cup EHC, Zajec J, S. VH. Occupational therapy guidelines CVA Arnhem and Nijmegen University of Applied Sciences and Occupational Therapy Netherlands2013 [cited 2022 February, 3]. Available from: <https://www.kennisnetwerkcva.nl/wpcontent/uploads/2018/08/Ergotherapierichtlijn-CVA-2013.pdf> |
| **NORWAY** |
| Norwegian Directorate of Health. National professional guideline for treatment and rehabilitation of stroke 2017 [updated 2020; cited 2022 February 3,]. Available from: <https://www.helsedirektoratet.no/retningslinjer/hjerneslag>. |
| **UK** |
| Intercollegiate Stroke Working Party. National Clinical Guideline for Stroke: Royal College of Physicians; 2016 [cited 2022 February, 3]. Fifth:[Available from: <https://www.strokeaudit.org/SupportFiles/Documents/Guidelines/2016-National-Clinical-Guideline-for-Stroke-5t-(1).aspx>. |
| National Clinical Guideline Centre (UK). Stroke Rehabilitation in adults UK: Royal College of Physicians 2013 [cited 2022 February, 3]. Available from: <https://www.nice.org.uk/guidance/cg162/resources/stroke-rehabilitation-in-adults-pdf-35109688408261>. |
| National Clinical Guideline Centre (UK). Stroke Rehabilitation: Long Term Rehabilitation After Stroke 2013 [cited 2022 February, 3]. Available from: <https://www.nice.org.uk/guidance/cg162/evidence/full-guideline-190076509> |
| Rudd AG, Bowen A, Young GR, James MA. The latest national clinical guideline for stroke. Clin Med (Lond). 2017;17(2):154-5. |
| Scottish Intercollegiate Guidelines Network. Management of Patients with Stroke: Rehabilitation, Prevention and Management of Complications, and Discharge Planning 2010 [cited 2022 February, 3]. Available from: <https://www.sign.ac.uk/media/1056/sign118.pdf>. |
| **EUROPEAN STROKE ORGANISATION** |
| Dziewas R, Michou E, Trapl-Grundschober M, Lal A, Arsava EM, Bath PM, et al. European Stroke Organisation and European Society for Swallowing Disorders guideline for the diagnosis and treatment of post-stroke dysphagia. European stroke journal. 2021;6(3):LXXXIX-CXV. |
| Norrving B, Barrick J, Davalos A, Dichgans M, Cordonnier C, Guekht A, et al. Action Plan for Stroke in Europe 2018-2030. Eur Stroke J. 2018;3(4):309-36. |
| Quinn TJ, Richard E, Teuschl Y, Gattringer T, Hafdi M, O’Brien JT, et al. European Stroke Organisation and European Academy of Neurology joint guidelines on post-stroke cognitive impairment. European Stroke Journal. 2021;6(3):I-XXXVIII. |
| **WORLD STROKE ORGANISATION** |
| Lindsay P, Furie KL, Davis SM, Donnan GA, Norrving B. World Stroke Organization global stroke services guidelines and action plan. Int J Stroke. 2014;9 Suppl A100:4-13. |
| **REGION: NORTH AMERICA (n= 14)** |
| **CANADA** |
| Cameron JI, O'Connell C, Foley N, Salter K, Booth R, Boyle R, et al. Canadian Stroke Best Practice Recommendations: Managing transitions of care following Stroke, Guidelines Update 2016. Int J Stroke. 2016;11(7):807-22. |
| Campbell C, Cayley M, Kapoor E, Lien. AVL, Swartz E. Stroke in Young Adults 2015 [cited 2022 February, 3]. Available from: <https://www.canadianstroke.ca/sites/default/files/resources/Stroke_Young_FINAL.pdf> |
| Casaubon LK, Boulanger J-M, Blacquiere D, Boucher S, Brown K, Goddard T, et al. Canadian Stroke Best Practice Recommendations: Hyperacute Stroke Care Guidelines, Update 2015. International Journal of Stroke. 2015;10(6):924-40. |
| Cotoi A, Batey C, Hussein Norhayati, Janzen S, Teasell R. Rehabilitation of Younger Patients Post Stroke, : Evidence-Based Review of Stroke Rehabilitation; [updated 2018; cited 2022 February, 3]. Available from: <http://www.ebrsr.com/sites/default/files/v18-SREBR-CH21-NET.pdf>. |
| Eskes GA, Lanctôt KL, Herrmann N, Lindsay P, Bayley M, Bouvier L, et al. Canadian Stroke Best Practice Recommendations: Mood, Cognition and Fatigue Following Stroke practice guidelines, update 2015. Int J Stroke. 2015;10(7):1130-40. |
| Hebert D, Lindsay MP, McIntyre A, Kirton A, Rumney PG, Bagg S, et al. Canadian stroke best practice recommendations: Stroke rehabilitation practice guidelines, update 2015. Int J Stroke. 2016;11(4):459-84. |
| Wein T, Lindsay MP, Côté R, Foley N, Berlingieri J, Bhogal S, et al. Canadian stroke best practice recommendations: Secondary prevention of stroke, sixth edition practice guidelines, update 2017. Int J Stroke. 2018;13(4):420-43. |
| **USA** |
| Billinger SA, Arena R, Bernhardt J, Eng JJ, Franklin BA, Johnson CM, et al. Physical activity and exercise recommendations for stroke survivors: a statement for healthcare professionals from the American Heart Association/American Stroke Association. Stroke. 2014;45(8):2532-53. |
| Management of Stroke Rehabilitation Working Group. VA/DOD Clinical practice guideline for the management of stroke rehabilitation. J Rehabil Res Dev. 2010;47(9):1-43. |
| Rodin M, Saliba D, Brummel-Smith K. Guidelines abstracted from the Department of Veterans Affairs/Department of Defense clinical practice guideline for the management of stroke rehabilitation. J Am Geriatr Soc. 2006;54(1):158-62. |
| Schwamm LH, Audebert HJ, Amarenco P, Chumbler NR, Frankel MR, George MG, et al. Recommendations for the implementation of telemedicine within stroke systems of care: a policy statement from the American Heart Association. Stroke. (2009) 40:2635–60. doi: 10.1161/STROKEAHA.109.192361 |
| Schwamm LH, Audebert HJ, Amarenco P, Chumbler NR, Frankel MR, George MG, et al. Recommendations for the Implementation of Telemedicine Within Stroke Systems of Care. Stroke. 2009;40(7):2635-60. |
| Winstein CJ, Stein J, Arena R, Bates B, Cherney LR, Cramer SC, et al. Guidelines for Adult Stroke Rehabilitation and Recovery: A Guideline for Healthcare Professionals From the American Heart Association/American Stroke Association. Stroke. 2016;47(6):e98-e169. |
| Wright L, Hill KM, Bernhardt J, Lindley R, Ada L, Bajorek BV, et al. Stroke management: updated recommendations for treatment along the care continuum. Intern Med J. 2012;42(5):562-9. |
| **REGION: SOUTH AMERICA (n= 1)** |
| **BRAZIL** |
| Associação Brasileira de Medicina Física e Reabilitação. Acidente Vascular Encefálico Crônico: Reabilitação: Associação Brasileira de Medicina Física e Reabilitação; 2012 [cited 2022 February, 3]. Available from: <https://amb.org.br/files/_BibliotecaAntiga/acidente_vascular_encefalico_cronico_reabilitacao.pdf> |
