# Supplementary material for: Age-specific information resources to address the needs of young people with stroke: a scoping review protocol
Source: Syst Rev. 2022 Dec 19;11:275. doi: 10.1186/s13643-022-02147-4 (PMC9761956; doi:10.1186/s13643-022-02147-4)
Supplement: Supplementary file 4 — Additional file 4: Appendix D. Stroke -support and stroke-related Non-Profit Organisations websites. [file 13643_2022_2147_MOESM4_ESM.docx]

**Appendix D. Stroke -support and stroke-related Non-Profit Organisations websites**

| **Organization** | **Location** | **Website** |
| --- | --- | --- |
| Associação AÇÃO AVC | Brazil | https://www.acaoavc.org.br/ |
| American Stroke Foundation | USA | https://americanstroke.org/after-stroke/ |
| BELIEVE - Stroke Recovery Foundation |  | https://believesrf.org/ |
| Bridges self-management | UK | <https://www.bridgesselfmanagement.org.uk/> |
| Different Strokes | UK | <http://www.differentstrokes.co.uk/> |
| Generation S Young Stroke Survivors |  | <http://www.orgsites.com/pa/generation-s/> |
| [Heart and Stroke Foundation of Canada](https://www.googleadservices.com/pagead/aclk?sa=L&ai=DChcSEwjptN3r3-71AhWYyJQJHRTfAW8YABAAGgJ5bQ&ohost=www.google.com&cid=CAESWOD2AZIefIS6itUeR4fOxrGVvq6-RqQervmYOdnI-Tnr6Rl8G07qTGY0EFBw84sOee3Zm_rnj8v5OV7gzt8zGxPo3lVlzlXw6XMvgM1w9ZmgnH4V9nMfCVE&sig=AOD64_19DLUpeud3bAwYI3MURIIlEQuflA&q&adurl&ved=2ahUKEwjtoM_r3-71AhXPjokEHfgCCpcQ0Qx6BAgDEAE) | Canada | <https://www.heartandstroke.ca/stroke/recovery-and-support/stroke-care/rehabilitation> |
| National Stroke Foundation Australia |  | https://strokefoundation.org.au/ |
| Stroke Association UK | UK | https://www.stroke.org.uk/ |
| Stroke Awareness and Support Association (SASA) |  | <http://strokeawarenessandsupport.com>  https://www.facebook.com/JoinSASA/photos/?ref=page_internal |
| Stroke Awareness Foundation |  | <https://www.strokeinfo.org/> |
| SameYou for Brain Injury Recovery | UK | <https://www.sameyou.org/> |
| Stroke Foundation | Australia | https://strokefoundation.org.au/ |
| Stroke Foundation New Zealand | New Zealand | https://www.stroke.org.nz/ |
| The Hazel K. Goddess Fund for Stroke Research in Women |  | <http://www.thegoddessfund.org/> |
| The Stroke Recovery Foundation |  | https://strokerecoveryfoundation.org/ |
| Young Stroke |  | <https://youngstroke.org/> |
