# Supplementary material for: Age-specific information resources to address the needs of young people with stroke: a scoping review protocol
Source: Syst Rev. 2022 Dec 19;11:275. doi: 10.1186/s13643-022-02147-4 (PMC9761956; doi:10.1186/s13643-022-02147-4)
Supplement: Supplementary file 5 — Additional file 5: Appendix E. Allied Health Professional Organizations. [file 13643_2022_2147_MOESM5_ESM.docx]

**Appendix E. Allied Health Professional Organizations**

| **National Physiotherapy Associations** | |
| --- | --- |
| Afghan Association for Physical Therapy | Lithuanian Physiotherapy Association |
| Albanian Association of Physiotherapists | Luxembourg Association of Physiotherapists |
| Argentine Association of Kinesiology | Macau Physical Therapists Association |
| Australian Physiotherapy Association | Association of Physiotherapists of Madagascar |
| Austrian Physiotherapy Association | Physiotherapy Association of Malawi |
| Bahamas Association of Physiotherapists | Malaysian Physiotherapy Association |
| Bahrain Physical Therapy Association | Association of Physiotherapists of Mali |
| Bangladesh Physiotherapy Association | Malta Association of Physiotherapists |
| Barbados Physical Therapy Association | Association of Physiotherapists (Mauritius) |
| Axxon, Physical Therapy in Belgium | Mexican Association of Physiotherapy |
| Beninoise Association of Physiotherapists Reeducators | Mongolian Physical Therapy Association |
| Bermuda Physiotherapy Association | Chamber of Physiotherapists of Montenegro |
| Bhutan Society of Physiotherapy | National Federation of Physiotherapists in Morocco |
| Association of Physiotherapists in Bosnia and Herzegovina | Myanmar Physiotherapy Association |
| Physiotherapists' Association of Brazil | Namibian Society of Physiotherapy |
| Bulgarian Association of Physiotherapists | Nepal Physiotherapy Association |
| Cambodian Physical Therapy Association | Royal Dutch Society for Physiotherapy |
| Cameroon Society of Physiotherapy | Physiotherapy New Zealand |
| Canadian Physiotherapy Association | Nigerien Physiotherapy Association |
| College of Physiotherapists of Chile | Nigeria Society of Physiotherapy |
| Colombian Association of Physiotherapy | Norwegian Physiotherapist Association |
| Congo Physical Therapists Union | Pakistan Physical Therapy Association |
| Physical Therapy Commission of the Therapist Guild of Costa Rica | Palestinian General Syndicate for Physical Therapy |
| Croatian Council of Physiotherapists | Panamanian Association of Physiotherapy and/or Kinesiology |
| Curacao Association of Physiotherapists | Papua New Guinea Physiotherapy Association |
| Cyprus Association of Physiotherapists | Peruvian Association of Physiotherapy |
| Union of Physiotherapists of the Czech Republic | Philippine Physical Therapy Association |
| Association of Danish Physiotherapists | Polish Chamber of Physiotherapists |
| Dominican Association of Physical Therapists | Portuguese Association of Physiotherapists |
| Ecuadorian Society of Physiotherapy | Puerto Rican Association of Physiotherapy |
| Estonian Association of Physiotherapists | Order of Physiotherapists in Romania |
| Eswatini Physiotherapy Association | Rwanda Physical Therapy Organisation |
| Ethiopian Physiotherapists' Association | Saudi Physical Therapy Association |
| Fiji Physiotherapy Association | Senegalese Association of Physiotherapists - Rehabilitators |
| Finnish Association of Physiotherapists | Singapore Physiotherapy Association |
| French National Council of Physiotherapists | Slovak Chamber of Physiotherapists |
| Georgian Association of Physical Therapy and Rehabilitation | Slovenian Association of Physiotherapists |
| German Association for Physiotherapy | South African Society of Physiotherapy |
| Ghana Physiotherapy Association | Spanish Association of Physiotherapists |
| Panhellenic Physiotherapists' Association | Sri Lanka Society of Physiotherapy |
| Guyana Physiotherapy Association | Physiotherapy Association of Saint Lucia |
| Haitian Physiotherapy Society | Sudanese Physiotherapy Association |
| Hong Kong Physiotherapy Association | Surinamese Association for Physiotherapy |
| Association of Hungarian Physiotherapists | Swedish Association of Physiotherapists |
| Icelandic Physiotherapy Association | physioswiss |
| Indian Association of Physiotherapists | Syrian Physical Therapy Association |
| Indonesian Physiotherapy Association | Taiwan Physical Therapy Association |
| Iranian Physiotherapy Association | Association of Physiotherapists in Tanzania |
| Irish Society of Chartered Physiotherapists | Physical Therapy Association of Thailand |
| Israeli Association of Physiotherapists | Togolese Physiotherapists' Association |
| Italian Association of Physiotherapists | Physiotherapy Association of Trinidad and Tobago |
| Ivorian Association of Masseurs-Kinesitherapists | Turkish Physiotherapy Association |
| Jamaica Physiotherapy Association | Uganda Association of Physiotherapy |
| Japanese Physical Therapy Association | Ukrainian Association of Physical Therapy |
| Jordanian Physiotherapy Society | Emirates Physiotherapy Society |
| Kenya Society of Physiotherapists | Chartered Society of Physiotherapy |
| Korean Physical Therapy Association | American Physical Therapy Association |
| Chamber of Physiotherapists of Kosovo | Physiotherapists' Association of Uruguay |
| Kuwaiti Physical Therapy Association | Venezuelan Federation of Physiotherapists |
| Latvian Physiotherapists' Association | Vietnam Physical Therapy Association |
| Order of Physiotherapists in Lebanon | Zambia Society of Physiotherapy |
| Physiotherapists' Association of the Principality of Liechtenstein | Zimbabwe Physiotherapy Association |
| **National Occupational Therapy Associations** | |
| Confederacion LatinoAmericana De Terapeutas Ocupacionales | Instituti Kosovar i Ergoterapise / Kosovo Institute of Ergotherapy |
| Occupational Therapy Africa Regional Group | Latvian Association of Occupational Therapists |
| Asia Pacific Occupational Therapy Regional Group | Lebanese Occupational Therapists Syndicate |
| Arab Occupational Therapy Regional Group | Lithuanian Association of Occupational Therapists |
| Asociacion Argentina de Terapistas Ocupacionales | Association Luxembourgeoise des Ergotherapeutes Diplomes |
| Armenian Ergotherapists Association | Macau Occupational Therapists Association |
| Occupational Therapy Australia | Association Malgache Pour L'Ergotherapie |
| Ergotherapie Austria | Occupational Therapy Association of Malawi |
| Occupational therapy Association of the Bahamas | Malaysian Occupational Therapy Association |
| Bangladesh Occupational Therapy Association | Malta Association of Occupational Therapists |
| Association of Caribbean Occupational Therapists | Occupational Therapists' Association (Mauritius) |
| Federation Nationale Belge des Ergotherapeutes | Asociacion de Profesionales en Terapia Ocupacional |
| Bermuda Occupational Therapy Association | Mongolian Occupational Therapy Association |
| Association of Occupational Therapists in Bosnia and Herzegovina | Occupational Therapy Association of Morocco |
| Botswana Occupational Therapy Association | Namibian Association of Occupational Therapists |
| Associacao Brasileira dos Terapeutas Ocupacionais | Ergotherapie Nederland |
| Association of Bulgarian Ergotherapists | Occupational Therapy New Zealand \| Whakaora Ngangahau Aotearoa |
| Canadian Association of Occupational Therapists | Occupational Therapist Association of Nigeria |
| Colegio de Terapeutas Ocupacionales de Chile | Norsk Ergoterapeutforbund |
| China Occupational Therapy Association | Pakistan Occupational Therapy Association |
| Colegio Colombiano de Terapia Ocupacional | Palestinian Association for Occupational Therapy |
| Asociacion de Profesionales en Terapia Ocupacional | Asociacion de Terapeutas Ocupacionales de Panama |
| Council of Occupational Therapists for the European Countries | Asociacion Profesional de Terapeutas Ocupacionales del Peru |
| Hrvatska udruga radnih terapeuta | Philippine Academy of Occupational Therapists Inc. |
| Cyprus Association of Occupational Therapists | Polish Occupational Therapy Association |
| Czech Association of Occupational Therapists | Associacao Portuguesa de Terapeutas Ocupacionais |
| Danish Association of Occupational Therapists | Professional Association of Occupational Therapists from Romania |
| Dominican Republic Society of Occupational Therapy | Russian Professional Association of Ergotherapists |
| Estonian Association of Occupational Therapists | Rwanda Occupational Therapy Association |
| Ergoterapeutfelagid / Faroese Occupational Therapists Association | Saudi Occupational Therapy Association |
| Suomen Toimintaterapeuttiliitto ry | Association of Occupational Therapists of Serbia |
| Association Nationale Francaise des Ergotherapeutes | Seychelles Occupational Therapy Association |
| Georgian Occupational Therapists' Association | Singapore Association of Occupational Therapists |
| German Association of Occupational Therapists | Slovenian Association of Occupational Therapists |
| Occupational Therapy Association of Ghana | Occupational Therapy Association of South Africa |
| Hellenic Association of Ergotherapists | Asociacion Profesional Espanola de Terapeutas Ocupacionales |
| Guyana Association of Occupational Therapists | Sri Lanka Society of Occupational Therapists |
| Haitian Association of Occupational Therapists | Swedish Association of Occupational Therapists |
| Hong Kong Occupational Therapy Association | ErgotherapeutInnen - Verband Schweiz |
| Icelandic Occupational Therapy Association | Taiwan Occupational Therapy Association |
| All India Occupational Therapists Association | Tanzania Occupational Therapy Association |
| Indonesian Occupational Therapists Association | Occupational Therapists Association of Thailand |
| Iranian Occupational Therapy Association | Trinidad and Tobago Occupational Therapy Association |
| Association of Occupational Therapists of Ireland | Association Tunisienne D'Ergotherapie |
| Israeli Union of Occupational Therapists | Turkish Ergotherapy Association |
| Associazione Italiana dei Terapisti Occupazionali | Uganda Association of Occupational Therapists |
| Occupational Therapy Association of Jamaica | Ukrainian Society of Ergotherapists |
| Japanese Association of Occupational Therapists | Royal College of Occupational Therapists |
| Jordanian Society for Occupational Therapy | American Occupational Therapy Association Inc |
| Kazakhstan Ergotherapy Association | Federacion Venezolana de Terapeutas Ocupacionales (Venezuela) |
| Kenya Occupational Therapists Association | Zambia Occupational Therapy Association |
| Korean Association of Occupational Therapists | Zimbabwe Association of Occupational Therapists |
| **National Speech And Language Therapy Organisations** | |
| European Speech And Language Therapy Association | Israeli Speech, Hearing and Language Association |
| International Association of Communication Sciences and Disorders | Federazione Logopedisti Italiani |
| Asociacion Argentina de Logopedia | Japanese Association of Speech Language-Hearing Therapists |
| Speech Pathology Australia | Association Luxembourgeoise des Orthophonistes |
| Bundesverband Diplomierte Logopädinnen Österreich | Malaysian Association of Speech-Language and Hearing |
| Union Professionnelle des Logopèdes Francophones | The Association of Speech Therapists |
| Vlaamse Verening voor Logopedisten | Sociedad Mexicana de Audiologia y Foniatria |
| Federal Council of Speech-language Pathology | Nederlandse Verening voor Logopedie en Foniatria |
| Brazilian Society of Spech-language Pathology | New Zealand Speech-language Therapists' Association |
| Speech-Language & Audiology Canada | Nigerian Speech and Hearing Association |
| Colegio de Fonoaudiólogos de Chile | The Norwegian Association of Speech and Language Therapists |
| Beijing Association of Voice | Colegio Nacional de Fonoaudiólogos de Panamá |
| The Hong Kong Association of Speech Therapists | Philippine Association of Speech Pathologists |
| Asociación Colombiana de Fonoaudiología y Terapia del Lenguaje | Polish Logopaedic Society |
| Asociación Costarricense de Terapeutas del Lenguaje | Associação Portuguesa Terapeutas da Fala |
| Asociacion de Linguistas de Cuba | Organización Puertorriqueña de Patología del Habla, Lenguaje y Audiología |
| Cyprus SLT-SLP Association | Association of Phoniatricians and Speech Therapists |
| The Association of Clinical Logopedics | Saudi speech Pathology and Audiology Association |
| Audiologopaedisk Forening | Speech Language Hearing Association, Singapore |
| Egyptian Society of Phoniatrics and Logopedics | South African Speech Language Hearing Association |
| Estonian Logopedists Union | Korean Academy of Speech-Language Pathology & Audiology |
| Suomen Puheterapeuttiliitto | Association Espanola de Logopedia Foniatria y Audiologia |
| Federation Nationale des Orthophonistes | The Swedish Association of Logopedists |
| Deutscher Bundesverband für Logopädie e.V. | Conference des Associations Professionnelles Suisses des Logopedistes |
| Panhellenios Syllogos Logopedikon | The Speech-Language-Hearing Association of the Republic of China |
| Ungarishe Gesellschaft fur Phonetik, Phoniatrie und Logopadie | Thai Speech and Hearing Association |
| The Icelandic Association of Speech Therapists and Speech-language Pathologists | Association of Speech and Language Pathologists |
| Indian Speech and Hearing Association | American Speech-Language-Hearing Association |
| Indonesian Speech Therapist Association | Asociación de Fonoaudiología del Uruguay |
| Royal College of Speech and Language Therapists | Federacion Latino-Americana de Sociedades de Foniatria Logopedia y Audiologia |
| Irish Association for Speech and Language Therapists |  |
